# A systematic review and meta-analysis of neuroimaging studies examining synaptic density in individuals with psychotic spectrum disorders

**DOI:** 10.1186/s12888-024-05788-y

**Published:** 2024-06-19

**Authors:** Muhammad Omair Husain, Brett Jones, Usman Arshad, Stephanie H. Ameis, Giselle Mirfallah, Christin Schifani, Terri Rodak, Madina Aiken, Mudassar Shafique, Fatima Ahmed, Aristotle Voineskos, Muhammad Ishrat Husain, George Foussias

**Affiliations:** 1 Campbell Family Mental Health Research Institute, Centre for Addiction and Mental Health, Toronto, Canada; 2 Department of Psychiatry, Temerty Faculty of Medicine, University of Toronto, Toronto, Canada; 3 Pakistan Institute of Living and Learning, Karachi, Pakistan; 4 Division of Psychology & Mental Health, University of Manchester, Manchester, UK

**Keywords:** Synaptic density, Psychosis, Schizophrenia, Neuroimaging

## Abstract

**Background:**

Psychotic disorders have long been considered neurodevelopmental disorders where excessive synaptic pruning and cortical volume loss are central to disease pathology. We conducted a systematic review of the literature to identify neuroimaging studies specifically examining synaptic density across the psychosis spectrum.

**Methods:**

PRISMA guidelines on reporting were followed. We systematically searched MEDLINE, Embase, APA PsycINFO, Web of Science and The Cochrane Library from inception to December 8, 2023, and included all original peer-reviewed articles or completed clinical neuroimaging studies of any modality measuring synaptic density in participants with a diagnosis of psychosis spectrum disorder as well as individuals with psychosis-risk states. The NIH quality assessment tool for observational cohort and cross-sectional studies was used for the risk of bias assessment.

**Results:**

Five studies (k = 5) met inclusion criteria, comprising *n* = 128 adults (psychotic disorder; *n* = 61 and healthy volunteers; *n* = 67 and specifically measuring synaptic density via positron emission tomography (PET) imaging of the synaptic vesicle glycoprotein 2 A (SV2A). Three studies were included in our primary meta-analysis sharing the same outcome measure of SV2A binding, volume of distribution (V_T_). Regional SV2A V_T_ was reduced in psychotic disorder participants in comparison to healthy volunteers, including the occipital lobe (Mean Difference (MD)= -2.17; 95% CI: -3.36 to -0.98; *P* < 0.001 ), temporal lobe (MD: -2.03; 95% CI: -3.19 to -0.88; *P* < 0.001 ), parietal lobe (MD:-1.61; 95% CI: -2.85 to -0.37; *P* = 0.01), anterior cingulate cortex (MD= -1.47; 95% CI: -2.45 to -0.49; *P* = 0.003), frontal cortex (MD: -1.16; 95% CI: -2.18 to -0.15; *P* = 0.02), amygdala (MD: -1.36; 95% CI: -2.20 to -0.52, *p* = 0.002), thalamus (MD:-1.46; 95% CI:-2.46 to -0.46, *p* = 0.004) and hippocampus (MD= -0.96; 95% CI: -1.59 to -0.33; *P* = 0.003).

**Conclusions:**

Preliminary studies provide in vivo evidence for reduced synaptic density in psychotic disorders. However, replication of findings in larger samples is required prior to definitive conclusions being drawn.

**PROSPERO:**

CRD42022359018.

**Supplementary Information:**

The online version contains supplementary material available at 10.1186/s12888-024-05788-y.

## Summations

• Psychotic disorders have long been considered neurodevelopmental disorders where excessive synaptic pruning and cortical volume loss are central to disease pathology.

• Although there is growing academic interest in the role of synaptic density in the pathophysiology of major mental disorders, we identified only five studies examining synaptic density in individuals with psychotic disorders.

• Synaptic density was significantly lower in several brain regions including the anterior cingulate cortex, hippocampus, occipital, temporal, parietal and frontal cortices, in individuals with psychotic disorders relative to controls.

**Supplementary Information:**

The online version contains supplementary material available at 10.1186/s12888-024-05788-y.

## Considerations

• Preliminary studies provide in vivo evidence for the presence of reduced synaptic density in early course and chronic schizophrenia, however, further investigation in larger samples is required prior to definitive conclusions being drawn regarding changes in synaptic density in schizophrenia.

• Reduction in synaptic density was more significant in several brain regions of individuals with chronic schizophrenia when compared to healthy controls. Presently is difficult to draw definitive conclusions about whether synaptic density reduction is driving the disease or may be influenced by iatrogenic (e.g., pharmacotherapy, psychotherapy, neurostimulation), lifestyle (e.g., smoking, exercise, diet) or other environmental factors (e.g., stress, medical co-morbidity).

**Supplementary Information:**

The online version contains supplementary material available at 10.1186/s12888-024-05788-y.

## Introduction

Psychosis is one of the 20 leading causes of disability worldwide, affecting 29 million people [1]. Psychotic and schizophrenia spectrum disorders, including diagnoses of schizophrenia, schizoaffective disorder, schizophreniform disorder, brief psychotic disorder, delusional disorder, bipolar disorder with psychosis, and substance induced psychosis are associated with significant distress, poor global functioning, disability, and elevated risk of suicide [2]. Conceptualization of schizophrenia as a specific disease entity has evolved over the past decades, with many now considering this disorder a heterogenous syndrome that is part of the wider psychosis spectrum [3]. However, shared risk factors across psychotic disorders have led to the consideration of a shared common aetiological pathway underlying these conditions [4, 5]. First Episode Psychosis (FEP) occurs at a young age (usually early 20s) and is a critical period influencing the long-term course of the disorder. FEP (the first 2–5 years after illness onset) is characterized by repeated relapses, which cause distress as well as disruption of social and occupational functioning. Young adulthood is a critical time for early detection and individualized care to prevent the development of more severe illness and long-term disability [6]. There is a need to improve the accuracy of identifying individuals at risk of developing psychosis as psychotic disorders remain one of the leading causes of disability and contribute to significant morbidity and mortality worldwide.

Psychotic disorders have long been considered neurodevelopmental disorders resulting from excessive synaptic pruning during adolescence [7] where cortical volume loss is central to disease pathology [8]. This theory has been further supported by studies showing that the onset of cognitive symptoms preceding psychosis coincides with the biological process of pruning in adolescence/young adulthood [9]. Structural brain alterations have been reported in psychotic disorders over the past five decades and are thought to be reflective of changes in synaptic density that are postulated to be a consequence of excessive synaptic pruning [10]. Largely our understanding of the neurostructural changes across psychiatric disorders have been informed by evidence from post-mortem studies, indirect neuroimaging measurements (e.g., grey matter volume/grey matter density) and preclinical studies [11]. Young adulthood is a critical period of neurodevelopment characterized by synaptic reorganization (i.e., synaptic pruning and dendritic remodeling [12]). Synaptic pruning involves selection of synapses and drives the refinement and maturation of neural circuits from childhood into adolescence and early adulthood [13]. It is crucial to the enhancement of neuronal transmission and lays the foundation for the finely tuned circuitry that is needed to support higher-order cognitive skills, such as executive functioning and social cognitive processing [14].

The hypothesis that excessive synaptic pruning during late adolescence/young adulthood may drive the onset of psychosis [7] is further supported by converging post-mortem evidence. Analysis of individuals with psychotic disorders revealed reductions in neurite number, neuronal connectivity, dendritic arborization and spine density, and synaptic vesicle release [15, 16]. Synaptic volume reduction in psychotic disorders is also supported by meta-analytic evidence of 31 post-mortem studies describing reductions in cortical post-synaptic elements [17]. Synaptophysin is the gold standard histological biomarker for presynaptic density [18] (i.e., the total number of surviving synapses after synaptic pruning is completed during normal developmental process throughout adolescence [19]). Significant reductions in synaptophysin, have been shown in the hippocampus, frontal and cingulate cortices of individuals with schizophrenia [20]. Some less studied pre- and postsynaptic markers such as complexins, synapsins, rab3A, PSD-95 and synaptotagmin have also shown small reduction in individuals with schizophrenia (vs. control) in the same regions [20, 21]. Research supports that most significant loss in cortical grey matter volume occurs in adolescence and young adulthood in individuals with early psychosis, which temporally correlates with post-mortem findings of increased synaptic pruning [22]. Grey matter reduction has also been demonstrated in individuals with increased risk of developing a psychotic disorder (Clinical High Risk (CHR) for psychosis), with most consistent volume loss in the hippocampus, frontal cortex and cingulate cortex [23]. Reductions in brain volume in individuals with CHR have been found to correlate with peripheral markers of inflammation [24]. Brain volume reductions in CHR and first episode psychosis are hypothesized to represent loss of synaptic volume [24].

Genome-Wide Association Studies (GWAS) identified that the major histocompatibility complex (MHC) is the gene locus most strongly associated with psychosis risk, and various genes in this region are implicated in synaptic elimination [25, 26]. Variations in genes that encode synaptic proteins, including genes for the synaptic vesicle glycoprotein 2 A (SV2A), an established marker of synaptic density, have been implicated in the pathophysiology of schizophrenia [25, 27, 28]. Synaptic vesicle glycoproteins 2 (SV2) are essential in synaptic vesicle exocytosis and neurotransmitter release and are present in the membrane of synaptic vesicles [29, 30]. SV2A is the most monodispersed of three SV2 isoforms, ubiquitously present in essentially all active synapses and all neuron types in the brain making it a unique target to image synaptic vesicle density in the human brain in vivo [29–31]. SV2A density is strongly correlated with the cellular and regional distributions of synaptophysin [32], the gold standard histological biomarker for pre-synaptic density [18]. Association between schizophrenia and a common genetic variant in the SV2A gene region (1q21.2) has also been reported [33].

Findings from post-mortem studies [17] have been limited by the inability to control for confounding factors such as age effects [29], medication exposure [34–36] and co-morbid conditions [37]. These confounders may contribute to the reported synaptic alterations, making it difficult to establish a clear link between synaptic markers and the neurobiology of psychotic disorders. While post-mortem studies have provided valuable insights into the association between synaptic density loss and psychotic disorders, more conclusive evaluation of this association requires precise in vivo investigation methods. Regional synaptic density is traditionally estimated via stereology, immunohistochemistry, and electron microscopy [18]. Until recently, investigation of synapses and their dynamic changes in living subjects has been limited by the lack of a suitable in vivo imaging biomarker. However, positron emission tomography (PET) imaging radiotracers targeting SV2A are now considered the first-in-class non-invasive method to measure synaptic density in vivo in humans [32]. Two PET tracers have been developed for human use, [^18^F]SynVesT-1 and [^11^C]UCB-J, share the same precursor UCB-J [38–40] and show nearly identical [41], outstanding imaging properties [32, 42] (i.e., fast and high brain uptake, appropriate tissue kinetics, high levels of specific binding). [^18^F]SynVesT-1 and [^11^C]UCB-J have an excellent test-retest reproducibility of binding parameters [43, 44] (< 9% variability for [^18^F]SynVesT-1, < 10% for [^11^C]UCB-J) in humans. The tracers have been validated as specific and stable in vivo markers for synaptic density through: (1) strong specificity to SV2A [32] (co-localization with SV2A immunoreactivity), (2) sensitivity to synaptic loss [32], and (3) stability of binding parameters during task-induced brain activation [45]. SV2A PET has further been evaluated as safe not only in adults but youth as young as 15 years of age [46] and has been used in a range of conditions: psychosis, epilepsy, depression, anxiety, PTSD, Parkinson’s Disease, Alzheimer’s Disease [AD]) [47–51]. Initial studies strongly support that reductions in synaptic density are present in schizophrenia and early psychosis, and linked to symptoms [29, 52–54]. While outcome measures considering the arterial plasma content of the radiotracer, such as the volume of distribution (V_T_), tend to provide most accurate quantifications of SV2A binding, they require arterial blood draws throughout the scans. Therefore, other studies explored the use of a reference tissue, the centrum semiovale, for quantification of SV2A binding, providing binding potential (BP_ND_) as outcome measure [55]. In addition to novel synaptic density PET, advances in diffusion-weighted magnetic resonance imaging (MRI), such as the introduction of biophysically plausible models including the most widely used Neurite Orientation Dispersion and Density Imaging (NODDI) [56], represent another exciting avenue to elucidate the underlying microstructural abnormalities in psychotic-spectrum disorders [57].

There is growing academic interest in the role of synaptic density in the pathophysiology of major mental disorders. Although there have been recently published review articles and commentaries relating to synaptic density in neuropsychiatric disorder broadly and psychotic disorders more specifically [10, 19, 47, 49], we are unaware of a systematic review and meta-analysis of the current evidence base. In the present article we provide a qualitative and quantitative synthesis of data from neuroimaging studies examining synaptic density across the psychosis spectrum. To our knowledge this is the first systematic review and meta-analysis of literature on neuroimaging studies specifically examining synaptic density i.e., studies that were directly quantifying synaptic density in individuals across the psychotic spectrum.

## Method

We conducted the review in accordance with Preferred Reporting Items for Systematic Reviews and Meta-Analysis (PRISMA) guidelines [58]. The protocol was registered with PROSPERO (CRD42022359018). The completed review remains aligned with the original PROSPERO protocol in terms of search strategy, research questions and methodology. This review aimed to broadly evaluate all research studies that examine in vivo neuroimaging markers of synaptic density across the psychotic spectrum.

Main outcome: To determine whether available studies provide support for altered synaptic density in individuals with psychosis, schizophrenia, as well as clinical high risk, at-risk mental state, and ultra-high risk for psychosis compared to healthy controls.

Additional outcomes:

1. Associations between synaptic density and symptoms of psychosis.
2. Associations between synaptic density and measures of cognitive function.
3. Associations between synaptic density and measures of general function.

### Search strategy and study selection

A comprehensive search strategy was developed with a health sciences research librarian (TR). After testing, revising, and finalizing a core search strategy in MEDLINE, the librarian translated the approved strategy for use in the following bibliographic databases: MEDLINE, Embase, APA PsycINFO, Web of Science and The Cochrane Library. Databases were searched from year of inception to the date of the search, December 8, 2023. The strategy was comprised of three concepts combined using Boolean operators: (1) psychotic spectrum disorders (e.g. “psychosis”, “psychotic*”, “schizo*”); (2) neuroimaging (e.g. “neuroimag*”, “magnetic resonance”, “MRI”); (3) synapses (e.g. “synap*”, “presynap”, “postsynap*”). Each concept was queried using database-specific subject headings, natural language keywords, and advanced search operators. No study type or language limits were applied to the search strategy; dissertations and book chapters were excluded as publication types when possible. Please see supplementary material for complete search strategies (See supplementary material Table 1). These database searches were supplemented by (1) checking the references of included articles for any other potentially eligible studies we may have missed; (2) contacting the lead authors of all included papers to check if they have any other eligible published data (3) checking for any identified conference abstracts where lead authors were contacted to check if they have eligible data (4) checking for non-English publications, seeking translations by contacting the primary authors to determine if English versions were available or if feasible to use translation services, and (5) checking reference lists of relevant reviews.

Search results were uploaded to the web-based software platform Covidence to screen studies. Duplicates were removed and screening was done in two steps. Firstly, titles and abstracts were screened and studies not fulfilling the inclusion criteria were excluded by four independent reviewers (MA, MS, BJ, GM). Where it was uncertain if studies met inclusion criteria, they were retained for the next stage of screening. Secondly, full-text articles were screened out based on inclusion/exclusion criteria again by four independent reviewers (MS, FA, BJ, GM) and discrepancies were resolved through discussion with a senior reviewer (MOH).

### Eligibility criteria

Both published and grey literature were eligible, however, published studies were only included if they were original peer-reviewed articles or completed clinical trials. Articles reporting on studies were included provided that they met the following criteria: (1) included participants having a diagnosis of psychotic disorder, including schizophrenia, schizoaffective disorder, schizophreniform disorder, brief psychotic disorder, delusional disorder, bipolar disorder with psychosis and substance induced psychosis, or individuals considered to be at clinical high risk for psychosis (CHR) (i.e., constructs of CHR, ultra-high risk (UHR) for psychosis or at-risk mental state (ARMS)) and comparison with healthy control individuals; (2) all study types regardless of design (e.g., if the neuroimaging study was embedded in a clinic trial it could still be included; longitudinal and cross-sectional imaging studies could be included); and (3) in vivo neuroimaging studies of any modality that were directly measuring synaptic density. Individuals are classified as being at CHR if they meet at least one of the internationally established ultra–high-risk (UHR) inclusion criteria (brief limited intermittent psychotic symptoms [BLIPS], attenuated psychotic symptoms [APS], genetic risk and deterioration syndrome [GRD]), or basic symptoms [BS], according to diagnostic instruments such as the Comprehensive Assessment of at Risk Mental States, Structured Interview for Psychosis–Risk Syndromes, or Basel Screening Instrument for Psychosis (for UHR), or the Schizophrenia Proneness Instruments or Bonn Scale for the Assessment of Basic Symptoms (for BS) [59–63].We excluded studies in which the primary diagnosis of participants was not psychosis, schizophrenia, psychotic disorder or at-risk of psychosis (i.e., CHR, UHR, ARMS). Non-English studies were excluded due to lack of capacity to translate articles.

### Critical appraisal

Four researchers (MS, FA, GM, BJ) independently rated the methodological quality of all included studies, and conflicts were resolved by consensus with MOH. The NIH quality assessment tool for observational cohort and cross-sectional studies was used for the risk of bias assessment [64]. In 2013, NHLBI developed a set of tailored quality assessment tools to assist reviewers in focusing on concepts that are key to a study’s internal validity [64]. The tools were specific to certain study designs and tested for potential flaws in study methods or implementation.

### Data extraction

Data extraction was completed by four reviewers (MS, FA, BJ, GM). Conflicts were resolved by discussion between the two reviewers and the senior reviewer (MOH). We extracted data using a predetermined form including details about author, year, sample size, in vivo neuroimaging modality, inclusion and exclusion criteria, setting, population, control group condition, primary outcomes, and secondary outcomes.

### Synthesis of results

We qualitatively pooled the studies by in vivo neuroimaging modality, psychotic disorder investigated, assessment method of establishing synaptic density, and clinical outcomes. Aggregate data (as opposed to individual patient-level) was used for the present quantitative analyses. Descriptive statistics of study and participant characteristics were examined, and if studies were sufficiently homogeneous, they were included in meta-analyses. The difference in SV2A binding between individuals with psychosis spectrum and healthy controls, as quantified by volume of distribution (V_T_) (i.e., the ratio of the radiotracer concentration in the target tissue region to what is in the arterial plasma) [65], was the main outcome [66]. Where both partial volume corrected (PVC) and non-corrected data were reported, we included the non-PVC corrected data in our analysis. For the primary analysis, pooled effect sizes (ES) and confidence intervals were calculated from continuous data Mean Differences (MD) as we only included a single measure (V_T_) of synaptic density. Secondary analyses of other measures of synaptic density will also be conducted when sufficient data is available. When required, standard deviation (SD) was inputted, using the formula (SD = SEM * sqrt(N)). A fixed-effects model was used as there was little heterogeneity between studies. Heterogeneity was estimated using the I^2^ value (I^2^ values < 50% indicate low-to-moderate heterogeneity, whereas I^2^ > 50% indicate moderate-to-high heterogeneity). A p value < 0.05 (two-tailed) was taken as a significance level. The statistical analysis of the extracted data was conducted using Cochrane Review Manager (RevMan) 5.4.1. Given the limited number of studies included, we did not explore potential explanatory factors using sensitivity analysis/meta-regression.

## Results

**Fig. 1:**
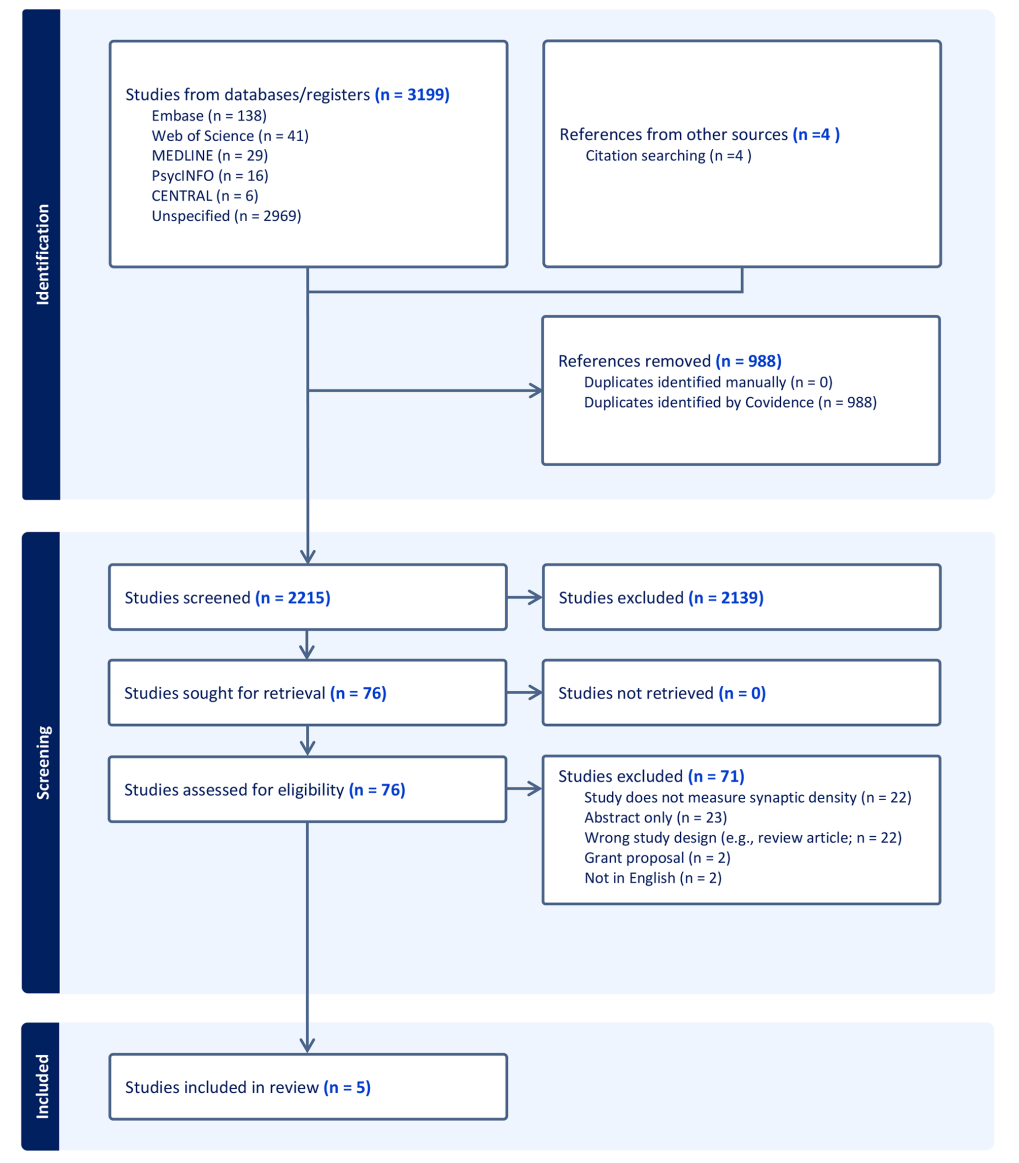
Prisma flow diagram

A total of 3199 studies were identified through a database search. An additional 4 studies were included from citation searches. We excluded 988 studies as they were duplicates. This left 2215 titles and abstracts that were screened and a further 2139 were excluded (Fig. 1). A full text review was completed on 76 articles. Of these another 23 were excluded as these were conference abstracts or supplementary. 2 studies were not in English and 2 were grant proposals. We contacted the corresponding authors and were unable to obtain English translated versions. We excluded 22 articles as they were not presenting original data and were either review articles or commentaries. We excluded 22 articles as they were not specifically measuring synaptic density, although they were neuroimaging studies within the target population. Four studies examined grey matter microarchitecture in individuals with chronic schizophrenia using neurite orientation dispersion and density imaging (NODDI) [67–70]. These studies were ultimately not included as they were not able to directly measure synaptic density.

### Study and sample characteristics

This systematic review identified 5 eligible studies in total (see Fig. 1 for PRISMA diagram) Study characteristics are summarised in Table 1. All five were cross-sectional studies using PET imaging with the [^11^C]UCB-J radiotracer in individuals with chronic and early-course schizophrenia (i.e., FEP). Three studies were conducted in the UK [52, 53, 71], and two in the United States [29, 54]. The studies comprised a total of *n* = 164 adults (i.e., participants over the age of 18 years) including *n* = 79 individuals with psychotic disorders and *n* = 85 healthy volunteers. The average age of the chronic and early course psychosis participants was approximately 40 and 28 years respectively, with a male predominance in the sample of over 80%. All chronic schizophrenia participants were treated with antipsychotic medications, either first or second generation, and the mean duration of illness was 17 + years. None of the early-course schizophrenia participants of the Onwordi et al., 2023 study were on antipsychotic medication during the duration of the study; they were all antipsychotic free for an average interval of 180.42 days with an average illness duration of 2.67 years. Early-course schizophrenia participants of the Yoon et al., 2023 study were on antipsychotic medication for an unspecified period with a mean duration of illness of 3.36 years.

**Table 1:** Study Characteristics of Cross-Sectional [^11^C] UCB-J PET Scan Studies

Author (Year), Country	Sample Size (n)	Patient Population	Age	Gender	Outcome Measure	Main Results
Onwordi et al. (2020), UK	Patients: 18 Controls: 18	Chronic SCZ	Patients: 41.5 (2.7) Controls: 38.7 (3.1)	Patients: 15 male and 3 female Controls: 15 male and 3 female	V_T_, DVR	• Mean [SEM] [^11^C]UCB-J V_T_ (ml/cm3) was significantly reduced in the SCZ relative to the HV group in the frontal cortex, FC (SCZ = 16.93 [0.80]; HV = 19.50 [0.64]; t = 2.51, df = 34.0, p = 0.03), and in the anterior cingulate cortex, ACC (SCZ = 19.55 [0.75]; HV = 22.49 [0.72]; t = 2.83, df = 34.0, p = 0.02), with large effect sizes (Cohen’s d = 0.8 and 0.9, respectively). • There were no significant relationships between [^11^C]UCB-J V_T_ in the FC, ACC or hippocampus, chlorpromazine-equivalent dose, Positive and Negative Syndrome Scale (PANSS) total, PANSS-positive, PANSS-negative or PANSS general scores or duration of illness • Mean (SEM) [^11^C]UCB-J DVR was significantly lower in the FC (SCZ = 2.93 [0.17]; HV = 3.48 [0.09]; *t* = 2.89, df = 34.0, *p* = 0.01, Cohen’s *d* = 1.0), ACC (SCZ = 3.39 [0.17]; HV = 3.99 [0.09]; *t* = 3.05, df = 34.0, *p* = 0.01, Cohen’s *d* = 1.0) and the hippocampus (SCZ = 2.40 [0.12]; HV = 2.74 [0.07]; *t* = 2.32, df = 34.0, *p* = 0.03, Cohen’s *d* = 0.8
Radhakrishnan et al. (2021), USA	Patients: 13 Controls: 15	Chronic SCZ	Patients: 40.52 (11.15) Controls: 40.77 (11.04)	Patients: 10 males and 3 females Controls: 12 males and 3 females	V_T_, BP_ND_	• Significant differences in the FC (% difference = − 10%, p = 0.01, Cohen’s d = 1.01), ACC (% difference = − 11%, p = 0.003, Cohen’s d = 1.24), hippocampus (% difference = − 15%, p = 0.002, Cohen’s d = 1.29), occipital cortex (% difference = − 14%, p = 0.001, Cohen’s d = 1.34), parietal cortex (% difference = − 10%, p = 0.03, Cohen’s d = 0.85), and temporal cortex (% difference= -11%, p = 0.003, Cohen’s d = 1.23). • Consistent across *BP*_ND_ and PVC BP_ND_, SCZ showed significantly lower synaptic density in the amygdala, fusiform gyrus, insula, pallidum, putamen, thalamus, and ventral striatum (range − 8–12%, Cohen’s *d* 0.8–1.2).
(Onwordi et al. (2021), UK	Patients:18 Controls: 22	Chronic SCZ	Patients: 40.89 (2.75) Controls: 38.23 (2.59)	Patients: 15 males and 3 females Controls: 21 males and 1 female	V_T_, DVR	• There were no significant relationships between [^11^C]UCB-J DVR and NAA/Cr in the hippocampus or ACC in schizophrenia patients.
(Onwordi et al. (2023), US	Patients: 21 Controls: 21	Early SCZ	Patients: 26.52 (1.74) Controls: 30.86 (1.90)	Patients: 17 males and 4 females Controls:16 males and 5 females	V_T_ and DVR, *V*_T_/*f*_p_	• There were no significant effects of group on [^11^C]UCB-J V_T_ or DVR in most regions of interest (effect sizes from d = 0.0–0.7, p > .05), with two exceptions that found lower distribution volume ratio in the temporal lobe (d = 0.7, uncorrected p < 0.05) and lower V_T_/*fp* in the ACC in patients (d = 0.7, uncorrected p < .05). The PANSS Positive and Negative Syndrome Scale total score was negatively associated with [^11^C]UCB-J V_T_ in the hippocampus in the SCZ group (r = − 0.48, p = .03). • DVR not significantly lower after FDR correction
Yoon et al. (2023), USA	Patients: 9 Controls: 9	Early SCZ	Patients: 25.67 (3.97) Controls: 27.22 (4.60)	Patients: 7 males and 2 females Control: 7 males and 2 females	BP_ND_	• Eight ROIs (left and right hippocampus, right superior temporal and Heschl's gyrus, left and right putamen, and right caudal and rostral middle frontal gyrus) showed large reductions. • Exploratory, atlas-wide analyses confirmed widespread reductions in schizophrenia. • It was also observed that there was a significant positive correlation between binding levels and cognitive performance and a negative correlation with the severity of delusions.

SEM: Standard Error Mean; SCZ: Schizophrenia; HV: Health Volunteer; PANSS: Positive and Negative Syndrome Scale; ROI: Regions of Interest; FC: Frontal Cortex; ACC: Anterior Cingulate Cortex; V_T_: Volume Distribution; DVR: Distribution Volume Ratio; BP_ND_ Binding potential; PVC: Partial Volume Corrected; fp: plasma-free fraction; FDR: False Discovery Rate; NAA/Cr: Creatine Scaled N-acetyl aspartate

### In vivo evidence of reduced synaptic density in schizophrenia

The seminal study examining synaptic density in vivo in psychotic spectrum disorders was led by Onwordi et al. (2020) [52]. They conducted two parallel studies. In their clinical PET study, they investigated volume of distribution (V_T_) of the [^11^C]UCB-J radiotracer, as a measure of synaptic density, in the frontal cortex (FC), anterior cingulate cortex (ACC) and hippocampus in participants with chronic schizophrenia (*n* = 18) and healthy controls (*n* = 18) [52]. The findings showed significantly lower [^11^C]UCB-J V_T_ in schizophrenia, compared to healthy volunteers, in the FC and ACC with large effect sizes (Cohen’s d > 0.8) [52]. Additionally, they showed evidence of lower V_T_ across multiple brain regions and that the hippocampus was less affected by synaptic density alterations than frontocortical regions [52]. Their parallel preclinical experiment investigated the effects of chronic antipsychotic drug exposure on SV2A levels and specific binding of [^3^H]UCB-J via autoradiography in drug-naïve rats. They found no significant effect on either measure concluding that antipsychotics are unlikely to impact the results of the clinical study in individuals with schizophrenia [52]. A subsequent study led by Radhakrishnan et al. (2021) also investigated SV2A binding in individuals with chronic schizophrenia (*n* = 13) and age- and sex-matched healthy controls (*n* = 15) using [^11^C]UCB-J PET imaging. They largely reproduced the initial findings of Onwordi et al. (2020) [29] when using binding potential (BP_ND_) and V_T_ as they outcome measures. V_T_ and BP_ND_ were significantly reduced in the schizophrenia participants compared to the healthy volunteer group in the ACC, FC, hippocampus, occipital, parietal and temporal cortices with large effect sizes (Cohen’s d = 0.85–1.34). Onwordi et al. (2021) completed a subsequent multi-modal imaging study (combining [^11^C]UCB-J PET with 1 H-magnetic resonance spectroscopy (1 H-MRS)) to test the relationship between synaptic density and glutamate levels in vivo, hypothesising that a negative relationship between the two existed in schizophrenia [71]. Although their primary findings indicated that synaptic density and glutamate levels were not related in schizophrenia, [^11^C]UCB-J V_T_ was significantly reduced in the left hippocampus and the ACC in participants with schizophrenia when compared with healthy controls [71]. Data from 17 of the 18 schizophrenia participants enrolled in this study and 17 of the 22 healthy volunteers were from their prior study. Onwordi et al. (2023) furthered their research by investigating the synaptic density in early course schizophrenia (i.e., FEP; *n* = 21) compared to healthy volunteers (*n* = 21) using [^11^C]UCB-J PET [53]. None of the FEP participants were taking antipsychotic medications, 2 were antipsychotic naïve and 19 had taken medication previously [53]. The results indicated that the SV2A levels measured with [^11^C]UCB-J V_T_ in untreated/minimally treated individuals early in the course of illness were not significantly different from demographically matched controls [53]. Yoon et al. (2023) conducted a similar study with a smaller sample size (*n* = 9 FEP vs. *n* = 9 controls) [54] using only BP_ND_ as their outcome measure (no arterial data acquired in this study). They reported a large reduction in all 8 selected ROIs, left and right hippocampus, right superior temporal and Heschl’s gyrus, left and right putamen, and right caudal and rostral middle frontal gyrus [54].

### Associations between synaptic density, symptom severity and cognition

Onwordi et al. (2020) examined the associations of synaptic density (via [^11^C]UCB-J V_T_) with symptom severity and cognitive performance and did not find a significant relationship [52]. Radhakrishnan et al. (2021) provided first reports of associations between regional reductions in synaptic density (via [^11^C]UCB-J V_T_) and disease characteristics in schizophrenia (i.e., symptom severity and cognitive performance) [29]. Symptom severity was measured using the positive and negative syndrome scale (PANSS) [72] and cognitive performance using the CogState Schizophrenia Battery [73] providing measures of speed of processing, attention/vigilance, working memory, visual learning, verbal learning, reasoning/problem solving, and social cognition, domains recommended by the MATRICS initiative [74]. Higher [^11^C]UCB-J V_T_ in the FC was correlated with reduced positive symptom severity but was not correlated with negative symptoms or general psychopathology [29]. Higher synaptic density in the FC was associated with better social cognition (i.e., better performance on emotion recognition tasks), and improved detection speed (assessed via neurocognitive processing tasks) [29]. Onwordi et al. (2023) reported a negative correlation between hippocampal [^11^C]UCB-J V_T_ and psychotic symptoms (PANSS scores) [53]. The recruited participants with early-course schizophrenia reported greater symptom severity than the chronic schizophrenia group recruited for the original Onwordi et al. (2020) study [53]. Yoon et al. (2023) utilized Brief Psychiatric Rating Scale (BPRS), Scale for the Assessment of Positive Symptoms (SAPS); Scale for the Assessment of Negative Symptoms (SANS), and the Brief Cognition in Schizophrenia (BACS) and found widespread positive correlations of [^11^C]UCB-J BP_ND_ with cognitive function and negative associations with symptom severity; i.e. better cognition associated with increased synaptic density and more severe delusions associated with lower synaptic density [54].

### Meta-analysis

Studies included in the meta-analysis were those that reported V_T_ measures. There was not sufficient data to conduct secondary analyses of other measures of synaptic density. Data from 17 of the 18 schizophrenia participants and 17 of the 22 healthy volunteers enrolled in the Onwordi et al. (2021) [71] study were from their prior study, therefore, we did not include it in our meta-analysis. Yoon et al. did not report V_T_ measure thus was not included in our primary analysis. Cochrane guidance indicates that minimum of two studies are required to proceed with meta-analysis [75]. Please see supplemental Table 2 for the complete list of reported measures of synaptic density (i.e., SV2A binding).

**Fig. 2:**
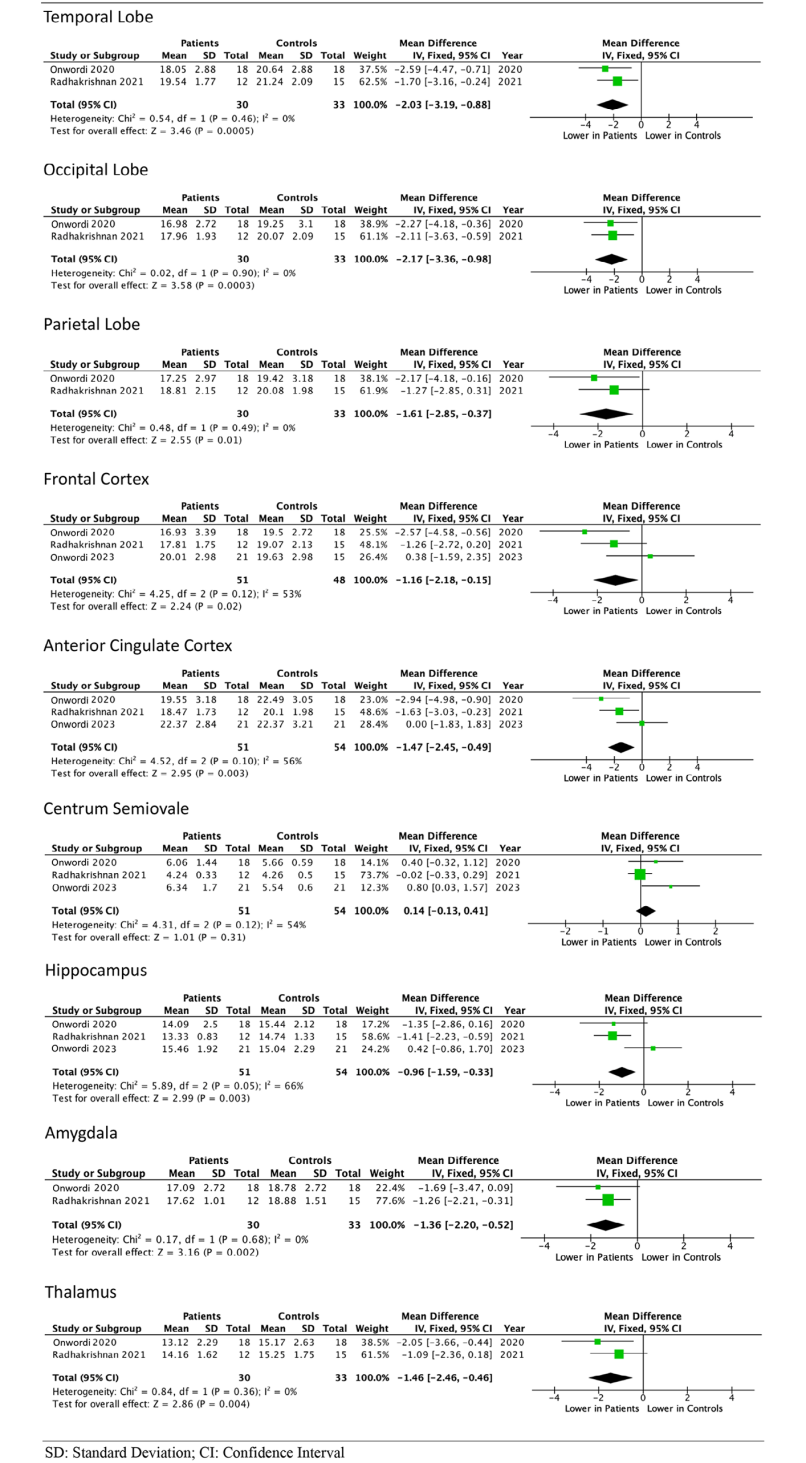
Forest Plots for Fixed Effects Meta-Analysis for Each Brain Region. SD: Standard Deviation; CI: Confidence Interval

Brain regions that showed a significant difference in synaptic density as measured by V_T_ between individuals with psychotic disorder and controls included the occipital lobe (MD= -2.17; 95% CI: -3.36 to -0.98; *P* < 0.001 ), temporal lobe (MD: -2.03; 95% CI: -3.19 to -0.88; *P* < 0.001 ), parietal lobe (MD:-1.61; 95% CI: -2.85 to -0.37; *P* = 0.01), anterior cingulate cortex (MD= -1.47; 95% CI: -2.45 to -0.49; *P* = 0.003), frontal cortex (MD: -1.16; 95% CI: -2.18 to -0.15; *P* = 0.02), amygdala (MD: -1.36; 95% CI: -2.20 to -0.52, *p* = 0.003), thalamus (MD:-1.46; 95% CI:-2.46 to -0.46, *p* = 0.004) and hippocampus (MD= -0.96; 95% CI: -1.59 to -0.33; *P* = 0.003). There was no significant difference in the centrum semiovale. Heterogeneity (I^2^) was 0% for the temporal lobe, occipital lobe, parietal lobe, thalamus, centrum semiovale, and amygdala. It was 53% for the frontal cortex, 56% for the anterior cingulate cortex, and 66% for the hippocampus (see Fig. 2. Forest plots).

### Sensitivity analysis

We completed random effects models for the three brain regions (frontal cortex, anterior cingulate cortex, and hippocampus) that reported moderate to high heterogeneity. When repeating the analysis, the directionality of the effect remained the same; however, the findings were no longer significant with the 95% CI crossing zero in all three cases.

### Critical appraisal of included studies

The NIH quality assessment tool for observational cohort and cross-sectional studies was used for the risk of bias assessment. Supplementary Table 3 summarizes the complete quality assessment. In terms of the hierarchy of evidence, our sample included 5 observational studies. Almost all studies clearly defined their study aim and the population they were recruiting. Sample size justification and power analysis was described in 2 out of the 5 studies. All studies used gold-standard approaches to examining synaptic density in the chosen populations (please see Table 2 and Supplementary Table 3). Two studies had a quality rating of good and 3 studies had a quality rating of fair.

**Table 2:** Risk of bias overall assessment

Author (Year), Country	Study Design	Clinical population	Comparator group	Sample size	Quality rating
Onwordi et al. (2020), UK	Cross Sectional	Chronic Schizophrenia	Healthy controls	36	Good
Radhakrishnan et al. (2021), USA	Cross Sectional	Chronic Schizophrenia	Healthy controls	28	Fair
(Onwordi et al. (2021), UK	Cross Sectional	Chronic Schizophrenia	Healthy controls	40	Fair
(Onwordi et al. (2023), US	Cross Sectional	Early- Course Schizophrenia	Healthy controls	42	Good
Yoon et al. (2023), US	Cross Sectional	Early- Course Schizophrenia	Healthy controls	18	Fair

## Discussion

This article set out to systematically review the published literature for in vivo neuroimaging studies specifically examining synaptic density in individuals across the psychosis spectrum and identified only five molecular imaging studies that included participants with chronic schizophrenia and early-course schizophrenia (i.e., first episode psychosis). All five PET studies included were cross-sectional in design, using a case-control model and were comprised of rather small sample sizes (18–42 participants). One of the five studies [71] recruited 17 of 18 participants from their prior published work [52]. There were no studies incorporating a longitudinal design. The molecular imaging studies included in our review demonstrated that SV2A PET radiotracer binding as measured by [^11^C]UCB-J V_T_ or BP_ND_ was significantly lower in several brain regions including the anterior cingulate cortex, hippocampus, occipital, temporal, parietal and frontal cortices, in individuals with chronic (3 out of 3 studies) and early-course (1 out of 2) schizophrenia relative to controls. In our meta-analysis all the regional [^11^C]UCB-J V_T_, with the exception of the centrum semiovale, were lower in schizophrenia participants in comparison to healthy volunteers. Although heterogeneity was low in the occipital lobe, temporal lobe, parietal lobe and hippocampus, there was greater heterogeneity in the frontal cortex, anterior cingulate cortex, and hippocampus [29, 53, 71]. When repeating the meta-analysis with a random effects model in the regions of higher heterogeneity, there was no longer a significant difference. Taken together, these studies provide preliminary evidence of synaptic density reduction in individuals with psychotic disorders though this is not consistently evident earlier in the course of the disease.

The purportedly central role of synaptic changes early in life and disease course to aetiology of psychosis was examined by two of the selected studies in this review. Onwordi et al. (2023) and Yoon et al. (2023) recruited participants with early-course schizophrenia, *n* = 21 and *n* = 9 respectively [53, 54]. Onwordi et al. (2023) found no significant difference in SV2A binding between early schizophrenia participants and healthy controls postulating that early in the course of disease synaptic density deficits maybe subtle becoming more apparent as illness progresses. On the other hand, Yoon et al., (2023) reported a widespread reduction in [^11^C]UCB-J binding potential (BP_ND_) in several brain regions including left and right hippocampus, right superior temporal and Heschl’s gyrus, left and right putamen, and right caudal and rostral middle frontal gyrus [54]. Data from the Onwordi et al. 2023 study were included in our meta-analyses. In the fixed effects models, the frontal cortex, anterior cingulate cortex and hippocampus showed significant differences compared to controls. The addition of the early course schizophrenia patients in these models; however, added significant heterogeneity and variability. Although these results are preliminary, they underscore the importance of investigating synaptic density across different stages of psychotic disorders and throughout the lifespan. This approach may provide deeper insights into the identification, progression, and treatment of these disorders.

This review emphasizes the importance of investigating the relationship between synaptic density and schizophrenia spectrum disorders. Radhakrishnan et al. (2021) provided first reports of correlations between regional reductions in synaptic density and disease characteristics in chronic schizophrenia where higher symptom severity was found to be associated with reduced cognitive performance. Onwordi et al. (2023) and Yoon et al. (2023) were able to replicate these findings in the early psychosis population where lower [^11^C]UCB-J binding correlated with greater symptom severity and reduced cognitive performance. These findings support the idea of excess in synaptic pruning during adolescence driving the disease rather than disease factors driving changes in synaptic density. While these findings are novel and speak to the potential clinical utility of measuring synaptic density as it relates to symptoms severity and cognition in individuals with schizophrenia, it is worth noting that these findings are based on exploratory analyses and warrant replication. Current pharmacotherapies are effective in improving positive symptoms but effects on negative symptoms and cognitive impairment, two domains have a significant impact on functioning and quality of life, are minimal at best [76, 77]. If negative symptoms and cognitive deficits are driven by decreased synaptic density, treatments that promote synaptic plasticity may address these disabling symptoms domains. There is a growing evaluation of novel therapeutics [78] that could have a role in modulating brain synapses [15].

Advances in diffusion-weighted MRI acquisition and modelling, including Neurite Orientation Dispersion and Density Imaging (NODDI) [56], have made it possible to model distinct aspects of grey matter microstructure related to neurites [79]. However, PET imaging studies targeting the SV2A receptor are the only neuroimaging modalities at present able to accurately quantify synaptic density in vivo. Four studies were included in full text review that employed the NODDI model and deserve mentioning. NODDI characterizes tissue into three compartments: intra-cellular, extra-cellular, and free-water providing indices of neurite density (neurite density index; NDI) and organization (orientation dispersion index; ODI) [57]. These indices allow the in vivo characterization of grey matter microstructure. NDI was reported to be reduced in the temporal, anterior parahippocampal and hippocampal regions of individuals with schizophrenia compared to healthy controls [69] which aligns with reductions in pre- and postsynaptic elements found in post-mortem studies [57]. In another study, ODI a putative marker of dendritic structure, was reported to be reduced in the anterior cingulate cortex and the medial frontal region in individuals with psychosis when compared with healthy controls [70]. Although NODDI indices are not presently considered proxies to synaptic density, they are promising tools with the potential to advance our understanding of the mechanisms underlying psychotic spectrum disorders, allowing direct insight into pathophysiological processes. They may be well positioned as biomarkers of psychiatric disease [80] given data acquisition being inexpensive, short (∼ 7 min) and employable on a larger scale as well as clinically. Additionally, recent evidence supports the correlation between ODI and SV2A binding [81]. Several studies to data have presented data on grey matter volume reductions in psychosis spectrum disorder individuals compared with health controls, however, the constituents of grey matter are thought to largely comprise of axons and dendrites, with synapses representing a much smaller component [10, 82].

### Strengths and limitations

This systematic review and meta-analysis drew on data from a limited pool of studies, which were of moderate quality. It is worth noting that there was greater heterogeneity in some brain regions, compared with others. It is unclear if the source of heterogeneity is driven by clinical heterogeneity in the schizophrenia population or secondary to methodological issues specifically related to small sample sizes in the included studies. Though it is worth noting that the only studies with elevated heterogeneity were those that included early course schizophrenia in their analysis. The SV2A PET imaging studies included in this review had several limitations. The small sample sizes of these preliminary studies suggest that further investigation is required prior to definitive conclusions being drawn regarding changes in synaptic density in schizophrenia. Although these studies provided preliminary in vivo evidence of synaptic reduction in schizophrenia, they were unable to control for smoking, antipsychotic use, and treatment effects in their patient samples [29, 52–54]. Notably, sex differences were also excluded in both analyses due to a small number of females in each study and the small total sample sizes [29, 52–54]. Although antipsychotic exposure was shown to have no effect on synaptic density in animal models and antipsychotics rarely bind to the SV2A receptors, it is possible that longer exposure to antipsychotic drugs could affect SV2A protein levels or specific binding [53, 54]. Taken together, the limitations of these studies make it difficult to draw conclusions about whether synaptic density changes are driving the disease or may be induced/influenced by iatrogenic (e.g., pharmacotherapy, psychotherapy, neurostimulation), lifestyle (e.g., smoking, exercise, diet) or other environmental factors (e.g., stress, medical co-morbidity). While acknowledging the difficulties of recruiting early-course schizophrenia subjects, enrolling participants with a shorter duration of illness and minimal/no antipsychotic exposure in future studies may provide greater insight into synaptic density changes in early-course schizophrenia. Additionally, PET scans are unable to capture spatial details in cortical layer 3 with an adequate resolution which could potentially obscure the SV2A levels [53, 54]. Furthermore, methodologically rigorous studies that have the adequate sample sizes and are appropriately powered to test the hypothesis that synaptic density is reduced in individuals with psychotic spectrum disorder are needed prior to more definitive conclusions being drawn.

## Conclusions

Psychotic disorders are associated with a significant degree of personal and societal burden globally. While morphometric studies support grey matter loss across the psychosis spectrum^34^ in vivo evidence of reduced synaptic density has only begun to emerge in psychotic disorders. To date, synaptic density in vivo has not been examined over time in schizophrenia to establish correlates with cognition, general functioning, and symptom severity change. Establishing the correlates of synaptic density with functioning, cognition, and symptom severity in psychotic disorders will shed light on neurobiological mechanisms of disease burden in these populations. Longitudinal PET studies have the potential to elucidate if changes in synaptic density relate to the temporal course of psychosis. Elucidating the mechanisms of symptom progression in psychotic disorders would have important clinical implications and the identification of illness markers could provide the opportunity for developing disease modifying interventions that may interrupt progression of disease. Identifying synaptic density as an endophenotype of psychosis would represent a milestone in psychosis research that could potentially lead to improved detection and personalized treatments for affected individuals. There is evidence for the modulation of synaptic plasticity with pharmacologic, neurostimulation and behavioral treatments [83, 84], however, synaptogenesis promoting interventions specifically targeting SV2A have yet to be established and could represent innovative treatment strategies for psychosis. Evaluation of synaptic density in individuals who are at clinical high risk for psychosis is also an area of future clinical research, which may provide further understanding of the neurobiological mechanisms of disease shedding light on synaptic density and how it relates to psychosis risk. Preventing psychosis and improving outcomes for affected individuals is a priority for health services across the world. Further investment is required to fund clinical research that aims to examine the role of synaptic density in the pathophysiology of psychosis spectrum disorders.

### Electronic supplementary material

Below is the link to the electronic supplementary material.

Supplementary Material 1: Search strategies

Supplementary Material 2: Complete list of measures

Supplementary Material 3: Quality assessment

## Data Availability

All data generated or analysed during this work are included in this published article and its supplementary information files.
