# Supplementary material for: A systematic review and meta-analysis of neuroimaging studies examining synaptic density in individuals with psychotic spectrum disorders
Source: BMC Psychiatry. 2024 Jun 19;24:460. doi: 10.1186/s12888-024-05788-y (PMC11188231; doi:10.1186/s12888-024-05788-y)
Supplement: Supplementary file 2 — Supplementary Material 2: Complete list of measures [file 12888_2024_5788_MOESM2_ESM.docx]

**Supplementary Materials Table 1: Search Strategies**

| Ovid MEDLINE: Epub Ahead of Print, In-Process & Other Non-Indexed Citations, Ovid MEDLINE® Daily and Ovid MEDLINE® <1946 to present> |
| --- |
| \| 1 \| Psychotic Disorders/ \| \| --- \| --- \| \| 2 \| exp Schizophrenia/ \| \| 3 \| affective disorders, psychotic/ \| \| 4 \| (psychosis or psychoses or psychotic*).ti,ab,kf. \| \| 5 \| schizo*.ti,ab,kf. \| \| 6 \| 1 or 2 or 3 or 4 or 5 \| \| 7 \| exp Neuroimaging/ \| \| 8 \| exp Magnetic Resonance Imaging/ \| \| 9 \| exp Functional Neuroimaging/ \| \| 10 \| exp positron-emission tomography/ \| \| 11 \| tomography, emission-computed/ \| \| 12 \| neuroimag*.ti,ab,kf. \| \| 13 \| neuro-imag*.ti,ab,kf. \| \| 14 \| magnetic resonance.ti,ab,kf. \| \| 15 \| MRI.ti,ab,kf. \| \| 16 \| positron-emission tomography.ti,ab,kf. \| \| 17 \| (PET adj3 scan*).ti,ab,kf. \| \| 18 \| 7 or 8 or 9 or 10 or 11 or 12 or 13 or 14 or 15 or 16 or 17 \| \| 19 \| exp Synapses/ \| \| 20 \| (synap* or presynap* or postsynap*).ti,ab,kf,hw. \| \| 21 \| 19 or 20 \| \| 22 \| 6 and 18 and 21 \| \| 23 \| exp animals/ not humans.sh. \| \| 24 \| 22 not 23 \| |

| Embase Classic+Embase |
| --- |
| \| 1 \| exp schizophrenia/ \| \| --- \| --- \| \| 2 \| exp psychosis/ \| \| 3 \| (psychosis or psychoses or psychotic*).ti,ab,kf. \| \| 4 \| schizo*.ti,ab,kf. \| \| 5 \| 1 or 2 or 3 or 4 \| \| 6 \| exp neuroimaging/ \| \| 7 \| exp nuclear magnetic resonance imaging/ \| \| 8 \| functional neuroimaging/ \| \| 9 \| exp positron emission tomography/ \| \| 10 \| neuroimag*.ti,ab,kf. \| \| 11 \| neuro-imag*.ti,ab,kf. \| \| 12 \| magnetic resonance.ti,ab,kf. \| \| 13 \| MRI.ti,ab,kf. \| \| 14 \| positron-emission tomography.ti,ab,kf. \| \| 15 \| (PET adj3 scan*).ti,ab,kf. \| \| 16 \| 6 or 7 or 8 or 9 or 10 or 11 or 12 or 13 or 14 or 15 \| \| 17 \| exp synapse/ \| \| 18 \| (synap* or presynap* or postsynap*).ti,ab,kf,hw. \| \| 19 \| 17 or 18 \| \| 20 \| 5 and 16 and 19 \| \| 21 \| (exp animal/ or nonhuman/) not exp human/ \| \| 22 \| 20 not 21 \| \| 23 \| exp schizophrenia/ \| \| 24 \| exp psychosis/ \| \| 25 \| (psychosis or psychoses or psychotic*).ti,ab,kf. \| \| 26 \| schizo*.ti,ab,kf. \| \| 27 \| 23 or 24 or 25 or 26 \| \| 28 \| exp neuroimaging/ \| \| 29 \| exp nuclear magnetic resonance imaging/ \| \| 30 \| functional neuroimaging/ \| \| 31 \| exp positron emission tomography/ \| \| 32 \| neuroimag*.ti,ab,kf. \| \| 33 \| neuro-imag*.ti,ab,kf. \| \| 34 \| magnetic resonance.ti,ab,kf. \| \| 35 \| MRI.ti,ab,kf. \| \| 36 \| positron-emission tomography.ti,ab,kf. \| \| 37 \| (PET adj3 scan*).ti,ab,kf. \| \| 38 \| 28 or 29 or 30 or 31 or 32 or 33 or 34 or 35 or 36 or 37 \| \| 39 \| exp synapse/ \| \| 40 \| (synap* or presynap* or postsynap*).ti,ab,kf,hw. \| \| 41 \| 39 or 40 \| \| 42 \| 27 and 38 and 41 \| \| 43 \| (exp animal/ or nonhuman/) not exp human/ \| \| 44 \| 42 not 43 \| |

| APA PsycInfo |
| --- |
| \| 1 \| exp psychosis/ \| \| --- \| --- \| \| 2 \| exp schizophrenia/ \| \| 3 \| (psychosis or psychoses or psychotic*).ti,ab,id. \| \| 4 \| schizo*.ti,ab,id. \| \| 5 \| 1 or 2 or 3 or 4 \| \| 6 \| exp neuroimaging/ \| \| 7 \| exp magnetic resonance imaging/ \| \| 8 \| positron emission tomography/ \| \| 9 \| exp tomography/ \| \| 10 \| neuroimag*.ti,ab,id. \| \| 11 \| neuro-imag*.ti,ab,id. \| \| 12 \| magnetic resonance.ti,ab,id. \| \| 13 \| MRI.ti,ab,id. \| \| 14 \| positron-emission tomography.ti,ab,id. \| \| 15 \| (PET adj3 scan*).ti,ab,id. \| \| 16 \| 6 or 7 or 8 or 9 or 10 or 11 or 12 or 13 or 14 or 15 \| \| 17 \| exp synapses/ \| \| 18 \| (synap* or presynap* or postsynap*).ti,ab,id,hw. \| \| 19 \| 17 or 18 \| \| 20 \| 5 and 16 and 19 \| \| 21 \| exp animals/ not humans.sh. \| \| 22 \| 20 not 21 \| \| 23 \| limit 22 to dissertation \| \| 24 \| limit 22 to chapter \| \| 25 \| 22 not (23 or 24) \| |

| Web of Science – ALL |
| --- |
| \| 1 \| psychosis or psychoses or psychotic* (Topic) \| \| --- \| --- \| \| 2 \| schizo* (Topic) \| \| 3 \| #1 or #2 \| \| 4 \| neuroimag* (Topic) \| \| 5 \| neuro-imag* (Topic) \| \| 6 \| "magnetic resonance" (Topic) \| \| 7 \| MRI (Topic) \| \| 8 \| "positron-emission tomography" (Topic) \| \| 9 \| PET NEAR/3 scan* (Topic) \| \| 10 \| #4 OR #5 OR #6 OR #7 or #8 or #9 \| \| 11 \| synap* or presynap* or postsynap* (Topic) \| \| 12 \| #11 AND #10 AND #3 \| \| 13 \| #11 AND #10 AND #3 \| \| 14 \| #11 AND #10 AND #3 and Book Chapters (Exclude – Document Types) \| \| 15 \| (Animal* or canine* or dog or dogs or feline* or hamster* or lamb* or mouse or mice or monkey* or murine or pig or pigs or piglet* or porcine* or primate* or rabbit* or rat or rats or rodent* or sheep or veterinar*) NOT (human* or patient*) (Title) \| \| 16 \| #14 NOT #15 \| |

| EBM Reviews - Cochrane Central Register of Controlled Trials  EBM Reviews - Cochrane Database of Systematic Reviews |
| --- |
| \| 1 \| (psychosis or psychoses or psychotic*).mp. \| \| --- \| --- \| \| 2 \| schizo*.mp. \| \| 3 \| 1 or 2 \| \| 4 \| neuroimag*.mp. \| \| 5 \| neuro-imag*.mp. \| \| 6 \| magnetic resonance.mp. \| \| 7 \| MRI.mp. \| \| 8 \| positron-emission tomography.mp. \| \| 9 \| (PET adj3 scan*).mp. \| \| 10 \| 4 or 5 or 6 or 7 or 8 or 9 \| \| 11 \| (synap* or presynap* or postsynap*).mp. \| \| 12 \| 3 and 10 and 11 \| \| 13 \| ((Animal* or canine* or dog or dogs or feline* or hamster* or lamb* or mouse or mice or monkey* or murine or pig or pigs or piglet* or porcine* or primate* or rabbit* or rat or rats or rodent* or sheep or veterinar*) not (human* or patient* or adult* or person* or people)).mp. \| \| 14 \| 12 not 13 \| |
